# L-Citrulline in Maternal–Fetal and Neonatal Health: Metabolic Mechanisms and Emerging Therapeutic Applications

**DOI:** 10.3390/nu18121923

**Published:** 2026-06-13

**Authors:** Ana Collins-Smith, Sangeeta Jain, Sunil Jain

**Affiliations:** 1 Division of Maternal-Fetal Medicine, Department of Obstetrics & Gynecology, The University of Texas Medical Branch at Galveston, Galveston, TX 77555, USA; sajain@utmb.edu; 2 Division of Critical Care Medicine, Department of Anesthesia, The University of Texas Medical Branch at Galveston, Galveston, TX 77555, USA; 3 Division of Perinatology, Department of Pediatrics, The University of Texas Medical Branch at Galveston, Galveston, TX 77555, USA; skjain@utmb.edu

**Keywords:** L-citrulline, arginine metabolism, nitric oxide bioavailability, maternal–fetal medicine, preeclampsia, intrauterine growth restriction, neonatal health, prematurity

## Abstract

L-citrulline is a non-protein amino acid with critical roles in perinatal and neonatal physiology through the intestinal–renal arginine–citrulline axis and nitric oxide (NO) production. During pregnancy, L-citrulline supports placental angiogenesis, vascular adaptation, and fetal growth through augmentation of arginine availability and endothelial NO production. In neonates, particularly preterm infants, developmental immaturity of citrulline and arginine synthesis contributes to hypoargininemia and may increase susceptibility to necrotizing enterocolitis, bronchopulmonary dysplasia with pulmonary hypertension, sepsis, and impaired intestinal function. Although L-citrulline has emerged as a promising modulator of NO bioavailability, prior reviews have largely focused on either adult cardiovascular disease or isolated neonatal applications, with limited integration of its mechanistic and translational relevance across the perinatal and neonatal continuum. Collectively, current evidence supports L-citrulline as a promising translational target in maternal–fetal and neonatal medicine because of its central role in vascular, inflammatory, and metabolic regulation. However, adequately powered clinical trials are needed to define optimal dosing, timing, patient selection, and long-term outcomes before routine clinical implementation can be recommended. This review provides a comprehensive evaluation of L-citrulline metabolism and its therapeutic potential from pregnancy through neonatal life, with emphasis on the intestinal–renal arginine–citrulline axis, endothelial function, and NO-mediated vascular regulation. We specifically examine the role of citrulline in key pathophysiologic mechanisms underlying maternal and neonatal disease, including endothelial dysfunction, impaired NO bioavailability, inflammation, oxidative stress, and abnormal placental vascular remodeling.

## 1. Introduction

L-citrulline is a non-protein amino acid that plays critical roles throughout the perinatal and neonatal periods. Named after the watermelon (*Citrullus vulgaris*) from which it was first isolated, citrulline serves as a key intermediate in the urea cycle and as the primary precursor for endogenous arginine synthesis [1]. Unlike most amino acids, citrulline is not incorporated into proteins and bypasses hepatic first-pass metabolism, making it an efficient vehicle for systemic arginine delivery [2].

The importance of citrulline in perinatal medicine has gained increasing recognition as research has elucidated its roles in placental vascular development, fetal growth, and neonatal adaptation. During pregnancy, adequate citrulline and arginine availability supports NO-mediated placental blood flow and angiogenesis [3]. In neonates, particularly those born prematurely, developmental limitations in citrulline metabolism contribute to arginine deficiency and associated complications [4].

The clinical importance of citrulline in maternal–fetal and neonatal medicine is underscored by the global burden of disorders associated with impaired placental vascular function, prematurity, and neonatal inflammatory disease. Preterm birth affects an estimated 13.4 million infants annually worldwide and remains the leading cause of death among children under five years of age [3]. Hypertensive disorders of pregnancy complicate approximately 2–8% of pregnancies globally and are major contributors to maternal and perinatal morbidity and mortality [3]. Additionally, necrotizing enterocolitis (NEC) is devastating complications of prematurity, affecting 5–10% of very low-birth-weight infants, and carries mortality rates approaching 20–30% in severe cases [4]. Emerging evidence suggests that impaired nitric oxide (NO) bioavailability, endothelial dysfunction, inflammation, oxidative stress, and abnormalities in arginine–citrulline metabolism may contribute to the pathophysiology of these conditions.

This review provides a comprehensive examination of citrulline’s role in perinatal and neonatal health, spanning from fundamental biochemistry to emerging clinical applications. In contrast to prior reviews that have focused primarily on isolated neonatal disorders or adult cardiovascular applications, this review integrates the evolving mechanistic, translational, and clinical evidence across the maternal–fetal–neonatal continuum.

### 1.1. Biochemistry and Metabolism of Citrulline

#### Citrulline Synthesis and Metabolic Pathways

Citrulline metabolism in mammals involves three distinct metabolic pathways distinguished by the tissue distribution of key enzymes [1]. First, in the liver, citrulline is locally synthesized by ornithine carbamoyltransferase (OCT) and metabolized by argininosuccinate synthetase (ASS) for urea production. Second, in most tissues producing NO, citrulline is recycled into arginine via ASS to increase arginine availability for NO production. Third, citrulline is synthesized in the gut from glutamine and released into the blood for conversion back to arginine in the kidneys—the intestinal–renal axis [1].

The primary site of citrulline synthesis is the enterocytes of the small intestine, where it is produced from glutamine, glutamate, and proline through the sequential action of pyrroline-5-carboxylate synthase and OCT [4]. During the neonatal period, de novo synthesis is the main source of ornithine for citrulline production, with proline serving as the preferred precursor [5]. Studies in neonatal piglets demonstrated that during fasting, plasma proline (13%) and ornithine (19%) were the main precursors for citrulline synthesis, while during feeding, enteral (27%) and plasma (12%) proline were the primary precursors [5].

### 1.2. The Intestinal–Renal Arginine–Citrulline Axis

The intestinal–renal axis for arginine synthesis represents a critical interorgan metabolic pathway, particularly important in perinatal and neonatal physiology. In this pathway, citrulline produced in the small intestine is released into the circulation and taken up by the kidneys, where it is converted to arginine through the sequential action of ASS and argininosuccinate lyase (ASL) [6].

Studies in neonatal pigs have demonstrated that this axis is present and functional from birth, despite the presence of ASS and ASL in the neonatal small intestine [6]. The lack of colocalization between citrulline-producing enzymes and citrulline-utilizing enzymes in the intestine results in net citrulline release from the gut and its subsequent utilization by the kidney to produce arginine [6]. This interorgan cooperation is essential for maintaining adequate arginine availability in neonates.

Additionally, important metabolic differences exist between term & preterm newborns that may influence the efficiency of this pathway. Compared with term infants, preterm neonates exhibit relative immaturity of intestinal epithelial function, mitochondrial oxidative metabolism, and renal enzymatic activity. Developmental studies have demonstrated that both preterm and term neonatal pigs have lower renal ASS1 and ASL expression compared with older animals, reflected by a prolonged citrulline half-life in neonatal groups [7]. However, these limitations are more pronounced in prematurity, where immature renal handling and reduced enzymatic capacity may contribute to impaired arginine generation and diminished NO bioavailability.

Mitochondrial maturation also plays a critical role in neonatal citrulline and arginine metabolism. During late gestation and the early postnatal transition, intestinal mitochondria undergo substantial structural and functional maturation to support increasing energy demands, oxidative phosphorylation, and amino acid metabolism. Preterm infants frequently demonstrate impaired mitochondrial maturation, reduced oxidative capacity, and altered substrate utilization, which may compromise enterocyte function and intestinal citrulline synthesis. Because enterocytes rely heavily on glutamine, glutamate, and proline metabolism for ATP production and citrulline generation, disruptions in intestinal energy metabolism may further impair the intestinal–renal axis in prematurity.

Consequently, strategies aimed at augmenting citrulline availability may provide therapeutic benefit not only by increasing arginine and NO production, but also by supporting mitochondrial function, improving endothelial homeostasis, and mitigating oxidative injury during critical stages of neonatal adaptation.

### 1.3. Citrulline and Nitric Oxide Production

The relationship between citrulline and NO production is of paramount importance in perinatal and neonatal physiology. Arginine serves as the substrate for nitric oxide synthase (NOS) enzymes, which produce NO and citrulline as a byproduct. This creates a metabolic cycle wherein citrulline can be recycled back to arginine, sustaining NO production [8].

Evidence suggests that citrulline may be a superior precursor for NO production compared to arginine itself. Citrulline supplementation leads to higher NO production because it bypasses hepatic first-pass metabolism, has high intestinal absorption and renal reabsorption, and prevents excessive and uncontrolled NO production [8]. At the cellular level, co-localization of citrulline transport systems and enzymes involved in the citrulline–arginine–NO pathway facilitates channeling of citrulline into NO production [8].

L-citrulline is more bioavailable than L-arginine because it avoids hepatic first-pass metabolism and has a longer circulation time [9]. This pharmacokinetic advantage makes citrulline particularly attractive as a therapeutic agent for conditions associated with NO deficiency.

### 1.4. Citrulline in Pregnancy and Fetal Development

#### Role in Placental Vascular Development

During pregnancy, the physiological adaptations are characterized by increased demands for L-arginine to sustain the growth of maternal, placental, and fetal tissues [10]. Although endogenous arginine synthesis is augmented during gestation and circulating concentrations of asymmetric dimethylarginine (ADMA)—an inhibitor of nitric oxide synthase (NOS)—are reduced, pregnancy remains a condition of relative arginine insufficiency [11]. This relative deficiency reflects a mismatch between supply and demand, particularly during periods of accelerated placental and fetal growth when arginine utilization exceeds both endogenous production and dietary intake [12]. A key phase of uteroplacental vascular development occurs between 8 and 20 weeks of gestation [13]. During this interval, cytotrophoblasts acquire an invasive phenotype, migrating into the decidua and remodeling maternal spiral arteries [14]. Through both interstitial and endovascular invasion, these trophoblasts convert the spiral arteries from high-resistance vessels into dilated, low-resistance channels capable of meeting the hemodynamic demands of pregnancy [15]. Nitric oxide, generated from L-arginine via endothelial nitric oxide synthase (eNOS), is integral to this process [16]. It mediates vasodilation, suppresses vascular smooth muscle proliferation, and enhances trophoblast invasion through effects on matrix remodeling enzymes and cell adhesion pathways [17].

Suboptimal arginine availability during this critical developmental window may impair angiogenesis and disrupt normal spiral artery transformation, contributing to uteroplacental insufficiency and increasing the risk of disorders such as preeclampsia and fetal growth restriction [18].

Citrulline supplementation offers a potential strategy to overcome these limitations by providing a more bioavailable precursor for arginine synthesis. In contrast to L-arginine, L-citrulline bypasses significant hepatic first-pass metabolism, resulting in improved systemic bioavailability [19]. Following absorption, it is converted to L-arginine in the proximal renal tubules via the argininosuccinate pathway, thereby providing a sustained substrate for NO synthesis [20].

Pharmacokinetic data indicate that oral L-citrulline supplementation produces a more sustained increase in circulating L-arginine concentrations compared with direct arginine administration [21]. Additionally, L-citrulline does not significantly stimulate arginase activity, thereby minimizing the diversion of arginine toward urea and ornithine production and preserving its availability for NO generation [22]. Its metabolic stability and role in nitrogen balance further support its utility within the urea cycle. Collectively, these characteristics position L-citrulline as a promising candidate for targeted supplementation in pregnancies affected by impaired NO bioavailability and aberrant placentation [22].

### 1.5. Intrauterine Growth Restriction

Intrauterine growth restriction (IUGR) results from either maternal undernutrition or impaired placental blood flow, exposing offspring to increased perinatal mortality and a higher risk of metabolic syndrome and cardiovascular disease during adulthood [23]. L-citrulline, as a precursor of L-arginine and NO, regulates placental blood flow and stimulates protein synthesis [23].

### 1.6. Preclinical Studies: IUGR

In a rat model of IUGR induced by maternal dietary protein restriction, L-citrulline supplementation (2 g/kg/day) increased fetal weight by approximately 9% compared to unsupplemented low-protein diet [23]. Fetal muscle protein fractional synthesis rate was 35% lower in IUGR fetuses and was normalized by L-citrulline supplementation [23]. Urinary nitrite and nitrate excretion, markers of NO production, increased in response to L-citrulline supplementation [23].

Further studies demonstrated that citrulline administered orally to the pregnant mother reaches fetal circulation, with maternal citrulline supplementation producing a 5-fold increase in fetal plasma citrulline and a 2-fold increase in fetal arginine [24]. Citrulline supplementation also upregulated the gene expression of several placental amino acid transporters, including SNAT4, LAT1, and LAT2 [24].

Maternal citrulline supplementation enhanced placental function and fetal growth through involvement of insulin-like growth factor 2 (IGF-2) and angiogenic factors [25]. The expression of Igf2-P0, a placenta-specific variant of the IGF-2 gene, and VEGF and Flt-1, involved in angiogenic pathways, was enhanced by citrulline supplementation [25].

Although most preclinical data derive from nutritional restriction models, findings from other experimental paradigms of placental insufficiency, including uterine artery ligation models, similarly support a role for impaired NO signaling and placental vascular dysfunction in the pathogenesis of IUGR. These observations further support the biologic rationale for citrulline supplementation as a potential therapeutic strategy to improve uteroplacental perfusion and fetal growth.

### 1.7. Clinical Studies: IUGR

Investigations from Poland by Rytlewski et al. reported reductions in fetal growth restriction alongside improved neonatal condition, as reflected by higher Apgar scores [26]. These results from this prospective clinical study, support the proposed mechanism whereby enhanced endothelial function and improved uteroplacental perfusion contribute to favorable fetal outcomes (even if supplementation initiated later in gestation; it conferred benefits).

In a randomized clinical trial, Neri et al. observed increased mean birth weights among individuals receiving L-citrulline, without evidence of treatment-related adverse events [27].

Camarena et al. demonstrated that L-citrulline supplementation in a prospective study was associated with a decreased incidence of preeclampsia, reduced rates of preterm birth, and increased neonatal birth weight [28]. These findings highlight its potential benefit in populations with a high prevalence of hypertensive disorders of pregnancy.

### 1.8. Preclinical Studies: Hypertensive Disorders of Pregnancy

Preclinical studies have demonstrated promising effects of citrulline in preeclampsia models. In Dahl salt-sensitive rats, a model of superimposed preeclampsia, L-citrulline supplementation significantly reduced maternal blood pressure, proteinuria, and levels of circulating soluble fms-like tyrosine kinase 1 (sFlt-1) [29]. L-citrulline improved maternal endothelial function by augmenting NO production in the aorta and improving endothelium-derived hyperpolarizing factor-mediated vasorelaxation in resistance arteries [29]. L-citrulline supplementation improved placental insufficiency and fetal growth, associated with enhanced angiogenesis and reduced fibrosis and senescence in the placentas [30].

Recent research has elucidated additional mechanisms by which citrulline improves preeclampsia pathophysiology. L-citrulline supplementation improves the IGF-1 signaling pathway in preeclampsia, at least partly via polyamine production [31]. In patients with preeclampsia, placental IGF-1 expression was significantly reduced compared with healthy pregnancy, and L-citrulline treatment maintained serum polyamine levels and normalized IGF-1 expression [31].

In a mouse model of preeclampsia, L-citrulline supplementation during pregnancy reduced blood pressure, increased vascular glycocalyx volume, and rescued ex vivo vascular function at late gestation [32]. The vascular benefits of L-citrulline extended postpartum, with improved vascular function and glycocalyx measures at 6 and 10 months of age [32].

### 1.9. Clinical Studies: Hypertensive Disorders of Pregnancy

The CITRUPE trial, a recent multicenter, randomized, double-blind clinical trial; found no benefit of oral L-citrulline supplementation in women with preeclampsia regarding duration of pregnancy, fetal growth, or maternal and neonatal outcomes [33]. Systolic blood pressure and liver enzyme levels were found to increase at delivery in the treated group, suggesting that L-citrulline oral supplementation may not be a promising therapeutic intervention in established preeclampsia [33].

In a separate randomized clinical trial, Dr. Powers and colleagues evaluated the effects of L-citrulline supplementation at a dose of 3 g/day in pregnant individuals with obesity, initiated at 16 weeks of gestation. The intervention was associated with improved maternal blood pressure profiles in the late third trimester and at the time of delivery. However, no sustained differences were observed in blood pressure measurements at six months postpartum, suggesting that the hemodynamic benefits of supplementation may be limited to the gestational period [34].

An early-phase randomized controlled trial conducted by Ormesher et al. examined the use of L-citrulline in pregnant patients with chronic hypertension. Participants received 3 g twice daily over an 8-week period beginning between 12 and 16 weeks of gestation. Although commonly reported pregnancy-related symptoms occurred in both study arms, the group receiving L-citrulline exhibited a lower incidence of preeclampsia. The investigators also reported increased rates of gestational diabetes and fetal growth restriction in the treatment arm; however, these findings may have been influenced by baseline differences, including a higher proportion of minority participants and female fetuses, both of which are independently associated with these outcomes [35].

Evidence from studies conducted outside of pregnancy further supports the safety and pharmacologic efficacy of L-citrulline. Dose-escalation trials in non-pregnant adult populations have demonstrated good tolerability at doses up to 10 g/day, with approximately 3 g/day identified as the threshold required to achieve meaningful increases in plasma L-arginine concentrations and nitric oxide production. Investigations by Schwedhelm, Moinard, and La Monica have consistently shown favorable pharmacokinetic profiles and improvements in vascular function [36]. Additional long-term safety data from Hafner et al. demonstrated that administration of 7.5 g/day over a 26-week period was well tolerated, with only mild gastrointestinal adverse effects reported in a minority of participants [28].

A systematic review and meta-analysis of L-arginine and L-citrulline for prevention and treatment of preeclampsia found that L-arginine was associated with a reduced risk of preeclampsia (RR 0.52; 95% CI, 0.35–0.78) and severe preeclampsia (RR 0.23; 95% CI, 0.09–0.55) in prevention trials [28]. However, only one study examined L-citrulline specifically and reported no effect on preeclampsia or blood pressure [28]. More trials are needed to determine the optimal dose and timing of supplementation.

In a large-scale trial conducted in Tanzania involving approximately 7000 pregnant participants, oral L-citrulline administration was associated with a reduction in the incidence of preeclampsia and improvements in neonatal outcomes [26]. The pragmatic design of the study emphasized feasibility and scalability, supporting the potential role of L-citrulline as an accessible intervention in low- and middle-income countries.

## 2. Materials and Methods

A comprehensive and systematic search of three major academic databases—PubMed (National Library of Medicine, Bethesda, USA), EMBASE (Elsevier, Amsterdam, Netherlands), and Web of Science (Clarivate, Philadelphia, USA)—was conducted through 1 April 2026, with no lower date restriction applied, ensuring the broadest possible capture of relevant historical and contemporary literature. No additional grey literature sources, conference abstracts, or book chapters were systematically searched; the review was intentionally scoped to peer-reviewed, indexed publications given the clinical and mechanistic focus of the topic.

Search strategies were tailored to the controlled vocabulary and indexing conventions of each database. PubMed searches employed Medical Subject Headings (MeSH) terms for “pregnancy” and “citrulline” as primary anchors, supplemented by free-text keyword searches. EMBASE searches used Emtree subject headings with an expanded set of pregnancy-related outcome terms, including miscarriage, stillbirth, congenital anomalies, hypertensive disorders of pregnancy, neonatal outcomes, and maternal complications. Web of Science, which does not use a controlled vocabulary, was searched using equivalent topic-field terms for both citrulline and pregnancy-related concepts to maximize coverage across disciplines. Across all databases, keyword searches included the following terms: citrulline, L-citrulline, arginine, nitric oxide, pregnancy, antepartum, postpartum, pregnancy complications, maternal complications, neonatal complications, maternal–fetal medicine, neonatal medicine, and perinatology.

Inclusion criteria were as follows: peer-reviewed articles published in English; studies involving female participants; studies pertaining to citrulline, L-citrulline, or related nitric oxide pathway substrates in the context of pregnancy, peripartum care, or neonatal outcomes.

Exclusion criteria included: case reports, given their limited generalizability; studies not available in English; (articles whose subject matter was determined upon full-text review to be irrelevant to the defined keywords or the thematic scope of this review (e.g., citrulline metabolism in non-pregnant adult populations with no translational relevance to pregnancy or perinatal medicine); and duplicate records across databases, which were removed prior to screening.

Following removal of duplicates, titles and abstracts were screened for relevance by the authors. Full texts of potentially eligible articles were then retrieved and assessed against the inclusion and exclusion criteria. The narrative, scope, and study type of each article were examined to determine its suitability for inclusion.

Given the broad and heterogeneous nature of the available literature, a narrative synthesis approach was adopted rather than a formal meta-analysis. Included studies were organized thematically according to the physiological and clinical subtopics addressed in this review—including citrulline’s role in the nitric oxide pathway, its relevance to placental function and vascular adaptation in pregnancy, its association with specific pregnancy complications (e.g., hypertensive disorders, fetal growth restriction), and its potential therapeutic applications in the perinatal period. Within each thematic section, evidence was synthesized by study design quality, mechanistic relevance, and clinical applicability, with higher-quality controlled studies highlighted where available and gaps in the literature noted explicitly.

### 2.1. Developmental Considerations in Neonatal Citrulline Metabolism

#### Enzyme Expression and Activity

Preterm infants face significant challenges in citrulline and arginine metabolism. The expression of key enzymes involved in arginine synthesis, including pyrroline-5-carboxylate synthase, ASS1, and ASL, is developmentally regulated and limited in preterm neonates [4]. Premature birth occurs before the normal perinatal surge of cortisol, which induces the expression of these key arginine-synthetic enzymes, thereby contributing to the hypoargininemia commonly observed in this population [4].

Studies in fetal and neonatal rats demonstrated that low citrullinogenesis (less than 60% of adult value) was observed throughout the suckling period when mitochondria isolated from newborn rat liver were incubated in vitro [37]. The adult value was reached only after weaning. The decreased citrullinogenesis seen for the first three days of life seemed to be related to the low intramitochondrial concentration of *N*-acetylglutamate, an activator of carbamoylphosphate synthetase-I [37].

### 2.2. Citrulline Levels in Preterm Infants

Plasma citrulline concentrations in healthy preterm infants show a progressive increase with postnatal age [38]. Reference intervals for plasma citrulline in children range from 13.31–69.05 μmol/L, though these values are widely dispersed [2]. In very low birth weight preterm infants receiving parenteral nutrition, median urinary citrulline levels of 24.7 μmol/mmol creatinine have been reported, with weak correlations to post-conceptional age and parenteral amino acid supply [38].

In preterm infants, urinary citrulline excretion correlates significantly with urinary nitrite and nitrate excretion, suggesting that one of the major determinants of urinary citrulline may be the biosynthesis of citrulline from arginine by NO-synthase [39]. Interestingly, urinary citrulline cannot be used to predict gastrointestinal tolerance in preterm infants, which is consistent with observations that in neonatal gut, citrulline is converted to arginine in situ rather than exported towards the kidneys as observed in adults [39].

### 2.3. Clinical Applications in Neonatal Morbidities

#### Necrotizing Enterocolitis

Necrotizing enterocolitis (NEC) is the most common gastrointestinal emergency in preterm infants with a mortality rate approaching 50% [40]. The pathogenesis of NEC is multifactorial involving: intestinal immaturity, impaired mucosal perfusion, exaggerated inflammatory signaling, microbial dysbiosis, and epithelial barrier dysfunction. NEC is associated with profound alterations in citrulline metabolism, and plasma citrulline levels are significantly lower in preterm neonates with NEC compared to age-matched controls, likely reflecting enterocyte injury [38]. As enterocytes are the principal site of endogenous citrulline production, declining citrulline levels may serve both as a marker of intestinal injury and as a contributor to impaired arginine and NO homeostasis.

### 2.4. Citrulline as a Biomarker

Plasma citrulline serves as a biomarker for enterocyte function and intestinal mass. In preterm neonates with NEC, mean citrulline levels were significantly lower compared to controls’ citrulline levels of the most approximate day of life [38]. The optimal citrulline cut-off distinguishing NEC patients from controls was 17.75 μmol/L (sensitivity 76%, specificity 87%) [38]. Plasma citrulline at presentation correlated inversely with the duration of parenteral nutrition [38].

Serial citrulline measurements during NEC show a progressive decrease in the first 48 h after onset, suggesting ongoing intestinal injury [41]. Citrulline levels measured in the first 24 h after NEC onset may provide an indication of intestinal recovery rate, with higher early levels associated with shorter time to full enteral feeding [41]. During recovery, plasma citrulline increases with reintroduction and gradual advancement of enteral nutrition [38].

Plasma citrulline was lower in infants with confirmed NEC (15.4 μmol/L) compared to those with NEC-like presentation (22.2 μmol/L) and healthy controls (24.9 μmol/L), suggesting potential utility in differentiating true NEC from other conditions presenting with similar symptoms [42].

### 2.5. Pathophysiological Role

Importantly, studies in preterm piglets have demonstrated that reduced citrulline–arginine–NO production precedes the onset of NEC rather than resulting from tissue damage [43]. Preterm pigs had lower citrulline production and arginine fluxes throughout the study period, with reduced gene expression in genes of the citrulline-arginine pathway preceding the development of NEC [43]. This suggests that impaired citrulline metabolism may be a predisposing factor for NEC development.

### 2.6. Supplementation Studies

Despite the pathophysiological rationale, recent evidence from a preterm pig model suggests that neither enteral nor intravenous supplementation of citrulline or arginine prevented NEC incidence or reduced its severity [40]. NEC incidence was highest in the control (60%), IV arginine (64%), and oral citrulline (62.5%) groups, while donor human milk showed a trend toward lower NEC incidence [40]. This indicates that while citrulline deficiency may contribute to NEC pathophysiology, supplementation alone may not be sufficient for prevention.

### 2.7. Bronchopulmonary Dysplasia and Pulmonary Hypertension

Bronchopulmonary dysplasia (BPD) is characterized by arrested alveolar development and is frequently complicated by pulmonary hypertension (PH). The pathophysiology of BPD-associated PH involves impaired angiogenesis, abnormal pulmonary vascular remodeling, endothelial dysfunction, chronic inflammation, oxidative stress, and disrupted nitric oxide (NO) signaling. NO is essential for normal pulmonary vascular adaptation after birth, mediating vasodilation, endothelial homeostasis, and alveolar-capillary development. Reduced NO bioavailability in premature infants may contribute to increased pulmonary vascular resistance, impaired vascular growth, and maladaptive remodeling of the pulmonary circulation. NO plays a crucial role in promoting alveolar growth, and plasma levels of NO precursors including citrulline are low in infants with pulmonary hypertension [44].

In the developing lung, endothelial dysfunction and chronic hypoxic exposure appear to play central roles in disease progression. Premature infants frequently experience intermittent hypoxia, hyperoxia, mechanical ventilation-associated injury, and inflammation, all of which disrupt endothelial NO synthase (eNOS) activity and promote pulmonary vasoconstriction. These abnormalities contribute to smooth muscle proliferation, increased pulmonary arterial medial wall thickness, and right ventricular strain. As the pulmonary vasculature & alveolar structures develop in close coordination during late gestation and early postnatal life, impaired vascular growth itself may directly contribute to arrested alveolarization. Consequently, therapies targeting endothelial function and pulmonary vascular signaling pathways may have important downstream effects on overall lung development.

### 2.8. Preclinical Evidence

Experimental studies in newborn rats exposed to hyperoxia (a model of BPD) have demonstrated that L-citrulline treatment significantly increases plasma arginine and citrulline concentrations, preserves alveolar and vascular growth, and decreases pulmonary arterial medial wall thickness and right ventricular hypertrophy [45]. L-citrulline also reverses the increased lung arginase activity observed in oxygen-exposed pups [45]. L-citrulline supplementation may represent a novel therapeutic alternative to inhaled NO for prevention of BPD [45].

More recent preclinical work has shown that L-citrulline attenuates lipopolysaccharide-induced inflammatory lung injury in neonatal rats by protecting against lung histopathology, reducing reactive oxygen species production, preventing NFκB nuclear translocation, and maintaining mitochondrial morphology and biogenesis [46]. L-citrulline maintained protein levels of PGC-1α, NRF1, and TFAM (transcription factors involved in mitochondrial biogenesis) and induced SIRT1, SIRT3, and superoxide dismutase protein expression [46]. These findings suggest that citrulline may decrease early lung inflammation and oxidative stress, potentially mitigating progression to BPD [46].

### 2.9. Clinical Studies

Pharmacokinetic studies in premature neonates at risk for BPD-associated pulmonary hypertension have characterized the disposition of enterally administered L-citrulline [47]. In ten premature neonates (≤28 weeks gestation) administered a single dose of 150 mg/kg oral L-citrulline, the volume of distribution (V/F) was 302.89 mL and clearance (CL/F) was 774.96 mL/h, with a half-life of 16 min (29-Fike). Simulated doses of 51.5 mg or 37.5 mg/kg given four times daily produced steady-state concentrations close to a target of 50 μmol/L [47].

Multi-dose enteral L-citrulline administration (60 mg/kg every 6 h for 72 h) has been evaluated in premature infants at risk for BPD-PH [30]. Target trough plasma L-citrulline concentrations were achieved in 2/6 subjects, and no serious adverse events occurred [48]. These results will assist in the design of phase II randomized controlled trials evaluating L-citrulline dosage strategies in infants at risk for BPD-PH [48].

Citrulline’s therapeutic potential in BPD extends beyond its role as an NO precursor. Citrulline may restore NO bioavailability, improve pulmonary vascular resistance, decrease right ventricular strain, and reduce the need for mechanical ventilation [44]. In BPD, citrulline could promote alveolar development, reduce inflammation, and improve long-term respiratory outcomes [44].

### 2.10. Neonatal Sepsis

The role of citrulline in neonatal sepsis represents a complex and evolving area of investigation with potentially contradictory findings depending on the clinical context, severity of illness, and developmental stage. While reduced citrulline availability may contribute to impaired endothelial function and diminished nitric oxide (NO) bioavailability during critical illness, excessive or dysregulated NO production may also exacerbate inflammatory injury and circulatory instability in advanced sepsis. These conflicting observations highlight the dual and context-dependent nature of the citrulline–arginine–NO pathway in neonatal immune responses.

### 2.11. Citrulline Metabolism in Sepsis

Sepsis profoundly disrupts citrulline metabolism in critically ill patients. Whole-body citrulline production is severely reduced in septic patients compared to healthy controls, with corresponding decreases in de novo arginine production and NO synthesis [49]. In adult septic patients, citrulline production was 4.5 ± 2.1 μmol·kg^−1^·h^−1^ compared to 13.7 ± 4.1 μmol·kg^−1^·h^−1^ in healthy controls [49]. This reduction in citrulline production is related to diminished conversion of glutamine to citrulline in the intestine [49].

Plasma citrulline concentrations are significantly lower in critically ill children with sepsis compared to those with viral respiratory disease, and these levels correlate strongly and inversely with severity of inflammation as measured by *C*-reactive protein [2]. Low plasma citrulline levels in severe sepsis have been associated with acute respiratory distress syndrome (ARDS), with ARDS rates of 50% in the lowest citrulline quartile compared to 15% in the highest quartile [49].

The immunologic mechanisms underlying these observations are multifactorial and involve complex interactions among endothelial dysfunction, immune cell activation, mitochondrial injury, oxidative stress, and dysregulated NO signaling. Under physiologic conditions, endothelial-derived NO supports vascular integrity, microcirculatory perfusion, leukocyte trafficking, and immune homeostasis. However, during severe sepsis, activation of inducible nitric oxide synthase (iNOS) within immune cells can result in excessive NO production, contributing to vasoplegia, hypotension, mitochondrial dysfunction, and nitro-oxidative stress. Thus, while reduced citrulline availability may impair protective endothelial NO signaling, uncontrolled inflammatory NO production may simultaneously promote tissue injury and organ dysfunction.

This apparent paradox highlights the growing importance of immunometabolism in neonatal sepsis. Immune cell activation during sepsis is accompanied by profound metabolic reprogramming, including shifts in mitochondrial oxidative phosphorylation, glycolysis, amino acid utilization, and redox balance. Arginine and citrulline metabolism are increasingly recognized as central regulators of these processes. Activated macrophages, neutrophils, and endothelial cells consume substantial amounts of arginine during inflammatory responses, potentially exacerbating arginine depletion in critically ill neonates with limited endogenous synthesis capacity. In preterm infants, developmental immaturity of mitochondrial function and antioxidant defense systems may further impair metabolic adaptation during sepsis.

### 2.12. Therapeutic Implications: An Age-Dependent Paradox

The therapeutic use of citrulline in sepsis presents a paradox, with divergent findings between adult/older animal models and preterm neonatal models.

In adult murine models of sepsis, citrulline administration has demonstrated beneficial effects on immune function. Citrulline supplementation was more efficient than arginine in increasing plasma arginine levels, restoring T cell mitochondrial function and proliferation, and reducing sepsis-induced regulatory T cell and myeloid-derived suppressor cell expansion [50]. Citrulline administration also markedly reduced immunosuppressive extrafollicular plasma cell differentiation and restored splenic follicles, potentially preventing secondary infections [51].

However, recent evidence from preterm piglet models reveals concerning opposite effects. In caesarean-delivered, parenterally nourished preterm pigs infected with Staphylococcus epidermidis, citrulline supplementation (1 g/kg bodyweight) exacerbated sepsis severity [52]. Oral citrulline supplementation led to higher mortality, increased blood bacterial load, and enhanced systemic and hepatic inflammation, while intravenous administration showed increased inflammation and bacterial burdens without significantly affecting mortality [34]. Liver transcriptomics and in vitro cord blood stimulation indicated that citrulline induces systemic immunosuppression in preterm newborns, which may impair the early resistance response to bacteria, subsequently causing uncontrollable inflammation and tissue damage [52].

This age-dependent paradox suggests that the immature immune system of preterm neonates may respond differently to citrulline supplementation compared to mature immune systems. The timing of supplementation relative to infection appears critical—citrulline may induce early immunosuppression that compromises bacterial clearance in the vulnerable preterm population [52].

## 3. Other Clinical Applications

A similar search strategy was conducted as described in Section 2, including the same databases, date range, controlled vocabulary terms, and inclusion and exclusion criteria. In addition to the primary search, supplementary searches were conducted to capture perinatal applications of citrulline that extend beyond maternal supplementation and antepartum complications. These additional searches examined citrulline’s role in neonatal and early postnatal contexts, including its use as a biomarker of intestinal function in preterm and critically ill neonates, its direct therapeutic administration to neonates (e.g., in the management of pulmonary hypertension of the newborn and urea cycle disorders), and its relevance to placental transfer and fetal substrate availability. Additional keywords used in these supplementary searches included: neonatal citrulline, citrulline biomarker, preterm infant, citrulline supplementation neonate, and postnatal citrulline.

### 3.1. Meconium Obstruction of Prematurity

Infants with meconium obstruction of prematurity demonstrate significantly decreased dried blood spot citrulline levels compared to controls, potentially indicating abnormal fetal intestinal development and reduced functional enterocyte mass [53]. This finding suggests that citrulline may serve as a biomarker for intestinal maturity and function in preterm infants.

### 3.2. Short Bowel Syndrome

Premature neonates with bowel resection have lower plasma citrulline concentrations than those without gastrointestinal disease, confirming citrulline’s role as a marker of gut mass [54]. Plasma citrulline concentrations correlate with residual small bowel length and may help predict intestinal adaptation and weaning from parenteral nutrition.

### 3.3. Urea Cycle Disorders

L-citrulline has been safely used from the neonatal period onwards in patients with urea cycle defects, particularly carbamoyl phosphate synthetase (CPS) and ornithine transcarbamylase (OTC) deficiencies, for decades [2,55]. In these conditions, citrulline supplementation bypasses the enzymatic defect and provides substrate for arginine synthesis, helping to maintain nitrogen balance and prevent hyperammonemia.

## 4. Safety and Pharmacokinetics of Citrulline Supplementation

### 4.1. Safety Profile in Neonates

In the limited human trials conducted to date, citrulline supplementation has demonstrated a favorable safety profile in stable preterm infants. A pilot randomized trial of 42 preterm infants (gestational age ≤33 weeks) receiving enteral L-citrulline at 100–300 mg/kg/day for 7 days reported no adverse events, with 95% of participants completing the supplementation period [56]. Plasma citrulline levels increased significantly in all three dose groups, while plasma arginine levels increased significantly in the high-dose group [56].

Similarly, early pharmacokinetic studies in extremely premature infants at risk for bronchopulmonary dysplasia-associated pulmonary hypertension did not identify clinically significant hypotension, feeding intolerance, or major laboratory abnormalities during short-duration enteral administration [47,48].

L-citrulline has been safely used from the neonatal period onwards in patients with urea cycle defects (CPS and OTC deficiencies) for decades [2]. Unlike direct arginine supplementation, citrulline prevents excessive and uncontrolled NO production while still supporting adequate NO synthesis [44]. Nevertheless, extrapolation from these chronic metabolic disorders to critically ill premature neonates should be approached carefully because premature infants possess fundamentally different metabolic, renal, hepatic, and immunologic physiology. Neonatal hepatorenal immaturity may alter citrulline handling, arginine generation, nitrogen balance, and NO production in ways that remain incompletely understood.

Importantly, although citrulline is often viewed as safer than direct arginine supplementation because it bypasses hepatic first-pass metabolism and may produce more regulated increases in NO synthesis, the long-term consequences of sustained modulation of the citrulline–arginine–NO pathway in developing neonates remain uncertain [44]. Potential concerns include altered vascular tone, disruption of oxidative-reductive balance, metabolic perturbations within the urea cycle, and immunologic effects related to dysregulated inflammatory signaling. Emerging experimental data have additionally suggested that excessive augmentation of NO pathways during severe inflammatory states may worsen immunosuppressive phenotypes or exacerbate sepsis-related vasoplegia in some neonatal models [51]. These conflicting observations underscore the need for careful disease-specific evaluation rather than assuming uniform benefit across clinical contexts.

### 4.2. Pharmacokinetic Considerations

The pharmacology of arginine/citrulline is confounded by several patient-specific factors such as variations in baseline arginine/citrulline due to developmental ages and disease states [9]. Currently available pharmacokinetic studies are insufficient to inform the optimal design of clinical studies, especially in children [9].

In premature neonates, L-citrulline exhibits a short half-life of approximately 16 min after enteral administration [29]. This rapid clearance necessitates frequent dosing to maintain target plasma concentrations. Simulated dosing strategies suggest that 37.5–51.5 mg/kg given four times daily may achieve steady-state concentrations near the target of 50 μmol/L [47].

Disease-specific physiology further complicates pharmacokinetic interpretation. In neonatal sepsis, systemic inflammation, mitochondrial dysfunction, altered microcirculatory perfusion, and increased inducible nitric oxide synthase (iNOS) activity may substantially alter citrulline utilization and NO dynamics. Similarly, in preeclampsia and placental insufficiency, maternal endothelial dysfunction and altered placental transport mechanisms may influence fetal citrulline and arginine availability. The impact of these pathophysiologic states on citrulline disposition remains poorly characterized and represents an important area for future investigation.

### 4.3. Cautions and Contraindications

Based on current evidence, citrulline supplementation should be avoided in neonates with active or suspected infection until further human safety data are available [51]. The dual role of NO in host defense and inflammatory injury raises concern that indiscriminate augmentation of the citrulline–arginine–NO pathway could potentially exacerbate vasoplegia, oxidative stress, or immune dysregulation in selected patients with sepsis.

Blood pressure monitoring is recommended during supplementation because of the theoretical risk of NO-mediated hypotension, particularly in extremely premature infants with limited cardiovascular reserve [28]. Careful consideration should additionally be given to renal and hepatic function, as organ immaturity may alter citrulline metabolism and nitrogen handling. Potential metabolic consequences of prolonged supplementation, including alterations in ammonia metabolism, amino acid balance, oxidative stress pathways, and mitochondrial function, remain insufficiently studied.

At present, the optimal dosing strategy, duration of therapy, target plasma concentrations, and long-term safety profile of citrulline supplementation in neonates remain undefined [44]. Larger randomized controlled trials incorporating pharmacokinetic modeling, developmental physiology, organ function assessment, and clinically meaningful outcomes will be essential before routine neonatal supplementation can be recommended.

### 4.4. Future Directions and Conclusions

L-citrulline represents a promising therapeutic agent for multiple conditions affecting perinatal and neonatal health, with particular relevance to conditions associated with nitric oxide (NO) deficiency, endothelial dysfunction, impaired angiogenesis, inflammation, mitochondrial injury, and oxidative stress [3]. Increasing evidence supports the central importance of the intestinal–renal arginine–citrulline axis during fetal and neonatal development, including in preterm infants, although developmental immaturity of intestinal, renal, and mitochondrial function may limit endogenous arginine production during periods of physiologic stress [7].

In pregnancy, citrulline supplementation shows promise for improving placental function and fetal growth in IUGR. Although studies evaluating established preeclampsia have yielded inconsistent results, emerging evidence suggests that earlier intervention during critical windows of placental vascular development may hold greater therapeutic promise [33]. Prevention trials with L-arginine have shown more consistent benefits, and further research is needed to determine whether citrulline offers advantages over arginine in pregnancy [28].

In neonates, current evidence supports the safety of citrulline supplementation in stable preterm infants and demonstrates efficacy in increasing plasma citrulline and arginine levels [28]. While citrulline shows promise in preclinical models of BPD and inflammatory lung injury and serves as a useful biomarker for intestinal function and NEC, further research is necessary to determine optimal dosing strategies and evaluate long-term efficacy in preventing or treating specific neonatal morbidities [38,45,46].

The emerging evidence regarding citrulline’s complex role in sepsis highlights the need for age-specific and context-specific investigation. The paradoxical immunosuppressive effects observed in infected preterm animals versus beneficial immune restoration in adult sepsis models underscore the importance of developmental stage in determining therapeutic responses [50,51,52]. Future studies must carefully consider the timing of supplementation relative to infection, the maturity of the immune system, and the specific clinical context.

Future investigations incorporating stable isotope tracer methodologies may further clarify citrulline flux, placental transfer, arginine generation, and nitric oxide kinetics across developmental stages and disease states.

Future randomized controlled trials should focus on establishing the clinical benefits of citrulline supplementation for preventing BPD-associated pulmonary hypertension, supporting intestinal recovery after NEC, and potentially reducing other complications of prematurity while carefully excluding or monitoring infants with active infections. The unique metabolic properties of citrulline, including its ability to bypass hepatic metabolism and preferentially support cellular NO production, position it as a potentially superior alternative to direct arginine supplementation in selected clinical scenarios in this vulnerable population.

Future investigations should also focus on pregnancy-specific and fetal-directed intervention strategies. However, advancement of therapeutic trials during pregnancy remains inherently challenging because pregnant individuals are classified as “therapeutic orphans,” resulting in substantial underrepresentation in drug development and clinical research. Concerns regarding fetal safety, liability, ethical complexity, and historically restrictive regulatory frameworks imposed by the U.S. Food and Drug Administration (FDA) have significantly limited interventional studies during pregnancy. Consequently, despite the biologic plausibility and promising preclinical data supporting L-citrulline supplementation in placental insufficiency and hypertensive disorders of pregnancy, adequately powered randomized clinical trials in pregnant populations remain limited.

To address the longstanding limitations of pregnancy research while maintaining compliance with evolving regulatory standards, alternative investigational approaches are increasingly being pursued in accordance with the FDA Modernization Act 2.0. These include advanced placental organoid systems, organ-on-chip technologies, ex vivo placental perfusion models, computational pharmacokinetic modeling, systems biology approaches, and translational animal models designed to better characterize placental transport, endothelial signaling, fetal exposure, and developmental safety. Such platforms may help bridge critical knowledge gaps while reducing reliance on traditional experimental paradigms that are difficult to implement in pregnant populations.

## Data Availability

The original contributions presented in this study are included in the article. Further inquiries can be directed to the corresponding author.
